# Development and validation of an ARID1A-related immune genes risk model in evaluating prognosis and immune therapeutic efficacy for gastric cancer patients: a translational study

**DOI:** 10.3389/fimmu.2025.1541491

**Published:** 2025-04-28

**Authors:** Jiangtao Zhang, Jingting Li, Shangfeng Yang, Xiaoyan Tang, Chunze Wang, Jiaxing Lin, Qiancheng Chen, Hui Xu, Yuanyuan Ma, Xiaoling Gao

**Affiliations:** ^1^ The Clinical Laboratory Center, Hainan General Hospital, Hainan Affiliated Hospital of Hainan Medical University, Haikou, Hainan, China; ^2^ Hainan Medical University, Haikou, Hainan, China; ^3^ Second Department of Critical Care Medicine, Xi’an Daxing Hospital, Shanxi, China

**Keywords:** ARID1A, gastric cancer, molecular docking, immune gene risk model, prognosis

## Abstract

**Background:**

Mutations in the ARID1A gene, an integral component of the SWI/SNF complex, are prevalent, affecting prognosis and immune response in several malignancies, including gastric cancer (GC). The aim of this study was to identify ARID1A mutation-associated immune genes to construct an ARID1A-related immune gene risk model (ARM).

**Methods:**

GSEA and ssGSEA were used to explore the involved biological pathways and the degree of immune cell infiltration, respectively. The prognosis model was constructed by lasso-COX. Protein expression level in tissue was verified by immunohistochemistry. Small molecule compounds were screened using molecular docking techniques and their anticancer value was validated *in vitro* and *in vivo* experiment.

**Results:**

This study revealed immune-related pathways and infiltration level of multiple immune cell types were enriched in the ARID1A^MUT^ group compared to the ARID1A^WT^ group. ARID1A mutations were correlated with an improved prognosis in individuals treated with immune checkpoint inhibitor (ICI) analyzed via Cbioportal website. TCGA-STAD cohort was randomly divided into a training-group and a testing-group. Additionally, ARM was developed in the training group, which identified APOD and PROC from ARID1A mutation-associated differential immunity genes. A significantly poorer prognosis in the high-risk group compared to the low-risk group, which was consistent across TCGA-training/testing/all cohorts, five GEO cohorts and 55 GC patients from Hainan General Hospital. Furthermore, the immune microenvironment components and ICI therapeutic efficacy markers were different between the two groups. Meanwhile, APOD and PROC expression was higher in GC tissues compared to para-cancerous tissues. Baicalin and capsaicin inhibited the proliferation and metastatic ability of GC cells.

**Conclusion:**

ARM provides valuable insights into the prognosis and the effectiveness of ICI, thereby offering a novel strategy for clinical decision. Baicalin and capsaicin are promising potential drugs for GC treatment.

## 1 Introduction

As extensively documented, epigenetic landscape alteration is among the hallmarks of human malignancies (1). As key players in epigenetics, chromatin remodeling factors, such as those in the mammalian SWI/SNF (mSWI/SNF) chromatin remodeling complex, modulate the expression of oncogenes and tumor suppressor genes, thereby playing pivotal roles in tumorigenesis. The SWI/SNF chromatin remodeling complex, driven by ATP hydrolysis, regulates nucleosome positioning and composition, thus maintaining cellular homeostasis and physiological functions. Exome sequencing studies have indicated that mutations in the SWI/SNF family occur in 19% of all cancer types, with a mutation rate of 36% in gastric cancer (GC), equivalent to that of TP53 (2). SWI/SNF family genes affect cancer progression by altering tumor cell behavior, modulating the expression of immune escape genes, and influencing components of the tumor microenvironment (TME) (3–5).

ARID1A (AT-rich interactive domain 1A, also referred to as BAF250) is the largest and most frequently mutated SWI/SNF subunit (2). Some drugs, such as ATM inhibitors, simvastatin and aspirin, act synergistically with PD-L1 inhibitors to inhibit tumor growth in ARID1A-deficient mice (6–8). ARID1A deletion results in the recruitment of T cells for anti-tumor immune responses through activation of the cGAS/STING pathway (9, 10). Studies have demonstrated that ARID1A mutations play a crucial role in the early onset, proliferation, metastasis, and prognosis of GC (11–13). Notably, in the context of tumor immunology, GC patients harboring ARID1A mutations exhibit higher levels of CD8^+^ T cell infiltration and derive greater benefit from immunotherapy. Mechanistically, ARID1A cooperates with STAT5 to promote the transcription of immunosuppressive factors such as TGF-β1 and NOX4, thereby influencing TME (14). Furthermore, ARID1A mutations are more frequently observed in molecular subtypes of GC that respond favorably to immune checkpoint inhibitors, such as microsatellite instability-high (15). ARID1A exerts dual functions in tumorigenesis. At the cancer cell-intrinsic level, ARID1A acts as a tumor suppressor by regulating the cell cycle, signaling pathways, and epithelial-mesenchymal transition (EMT). Conversely, from the tumor microenvironment perspective, ARID1A mutations shape an inflamed (hot) tumor phenotype, thereby enhancing sensitivity to immunotherapy. Given these findings, further investigation into ARID1A-associated immune-related genes is warranted to elucidate their role in shaping the immune landscape of GC aimed at optimizing clinical prognostic tools and enhancing immunotherapy efficacy.

The current study aimed to investigate the association between ARID1A mutations and the TME of GC and their prognostic value in an ICI-treated population using publicly available datasets. In the Cancer Genome Atlas-Stomach Adenocarcinoma (TCGA-STAD), an immune-related risk model (ARM) was constructed based on differentially expressed immune genes associated with ARID1A mutations. Next, five Gene Expression Omnibus (GEO) GC cohorts were used for external validation, and a retrospective study of 55 GC samples from our hospital further validated the prognostic value of ARM. Additionally, the potential of ARM as a biomarker for ICI treatment and the immune infiltration landscape linked to ARM was explored. Overall, ARM provides a novel strategy for assessing GC prognosis and optimizing ICI treatment selection. Furthermore, this study explores the potential of baicalein (Ba) and capsaicin (Ca) as therapeutic agents for GC through *in vitro* and *in vivo* experiments.

## 2 Materials and methods

### 2.1 Data source

RNA-seq data and clinical information for 375 GC cases were acquired from TCGA, along with supplementary data on 443 somatic mutations. TCGA-STAD transcriptome data were converted from fragments per kilobase million (FPKM) to transcripts per million (TPM). A subset comprising 366 STAD samples, with available mRNA expression data and somatic mutation information, was selected for further in-depth analysis. In addition, five microarray cohort studies of GC with available prognostic clinical data, namely GSE62254 (n = 297), GSE84437 (n = 431), GSE26942 (n = 202), GSE15459 (n = 182), and GSE26253 (n = 432), were retrieved from NCBI’s GEO database and integrated into the study. For duplicate gene names, average expression values were calculated.

RNA-seq data for the PD-L1 treatment cohort (IMvigor210) was sourced from [http://research-pub.gene.com/IMvigor210CoreBiologies]. The cBioPortal database [http://www.cbioportal.org/] was accessed to download mutation and prognosis data for 2,041 cancer patients who underwent immunotherapy (16, 17). Download data on 1,793 immune-related genes listed in the immunology database and analysis portal (IMMPORT) (18), accessible at [https://www.immport.org/home].

### 2.2 Enrichment and differential analysis

Relevant biological pathways were explored by conducting Gene Set Enrichment Analysis (GSEA) (19) using gene sets from the Gene Ontology (GO) and Kyoto Encyclopedia of Genes and Genomes (KEGG) databases. Differential expression analysis was performed using the “Limma” package on the 84 ARID1A mutation (MUT) and 282 wild-type (WT) cohorts. The criteria for identifying differentially expressed genes were set at an false discovery rate (FDR) below 0.05 and a minimum absolute log fold change (logFC) of 1.

### 2.3 Construction and validation of the immune gene-related risk (ARM) model

TCGA-STAD samples with less than 30 days of survival time were excluded, resulting in a differential immune gene expression matrix containing 337 samples. A 3:1 ratio was used to divide the TCGA-STAD dataset into the TCGA-train and test groups. In the TCGA-train group, immune genes with a univariate Cox P-value < 0.05 and |HR| > 1 were identified as prognostically significant. Following this, the least absolute shrinkage and selection operator (Lasso) COX regression analysis was subsequently carried out to further narrow down the shortlisted genes. Next, the “glmnet” R package was used to construct a multivariable Cox model. For each STAD sample, the following formula was applied to calculate the risk score:


$$\text{Risk score}=\sum_{\text{i}=1}^{\text{n}}\left({\text{Coefficient}}_{\text{i}}\times {\text{Expression}}_{\text{i}}\right)$$

Here, “n” represents the number of genes in the ARM, “expression_i_” denotes gene expression level, and “coefficient_i_” stands for the regression coefficient of each gene. The risk score formula was applied for both internal validation within the TCGA dataset and external validation using GEO cohorts. Based on the median risk score, survival curves were plotted using the Kaplan-Meier (KM) method. The time-dependent ROC analysis was performed to assess the sensitivity and specificity of ARM. The “tSNE” and “PCA” R packages were used to visualize the distribution of high-risk and low-risk populations. To evaluate the independent prognostic value of ARM risk scores, both univariate and multivariate Cox regression analyses were conducted.

### 2.4 Quantitative real-time polymerase chain reaction assay

Total RNA was extracted from cells using the Total RNA Mini Kit (Axygen, Shanghai, China) according to the manufacturer’s instructions. Complementary DNA (cDNA) was synthesized from the extracted RNA using the RevertAid First Strand cDNA Synthesis Kit (Thermo Fisher Scientific, Shanghai, China). The qPCR reaction mixture consisted of SYBR™ Green Master Mix (Thermo Fisher Scientific, Lithuania) cDNA template, nuclease-free water, and gene-specific primers. The mixture was aliquoted into eight-strip PCR tubes and subjected to amplification using a QuantStudio 5 PCR instrument (Applied Biosystems). The Ct values were recorded for each sample, and relative gene expression levels were calculated using the 2^^-ΔΔCt^ method. Primer sequences for the internal control (GAPDH) and target genes were shown in **Supplementary Table 1**.

### 2.5 Analysis of immune cell infiltration and immune response

The “GSVA” package (20) within the R programming environment was used to conduct ssGSEA, which facilitated the assessment of infiltration levels of multiple distinct immune cell types across each GC specimen. Additionally, the tumor immune dysfunction and exclusion (TIDE) server (http://tide.dfci.harvard.edu) was used to obtain information on the responsiveness of each TCGA-STAD sample to ICI therapy.

### 2.6 Immunohistochemistry staining

GC and matched paracancerous tissue samples were randomly selected from Hainan General Hospital. This study was approved by the Ethics Committee of our hospital and performed in accordance with ethical guidelines. Specific antibodies for IHC were purchased from Proteintech (APOD, 10520-1-AP, 1:100; PROC, 25382-1-AP, 1:50). After dewaxing, the tissue sections were baked at 60°C for 1h in a constant-temperature oven. Subsequently, the samples were sequentially immersed in xylene I-III for 10 minutes each, followed by immersion in anhydrous ethanol I–III for 10 minutes each. Then, the tissue sections were rinsed with running water for 12 minutes. Protein activity was inactivated using 3% H_2_O_2_ and a 1 mL NaN_3_ solution by soaking for 10 minutes, followed by washing with tap water for approximately 10 minutes. Antigen retrieval was performed using a pressure cooker. A 5% bovine serum albumin (BSA) blocking solution was applied and incubated for 35 minutes before incubation with the primary antibody overnight at 4°C. Subsequently, the sections were stained, dehydrated, and sealed. Finally, a semi-quantitative analysis was performed using Visiopharm software to determine the H-score for each specimen, which was calculated based on the number of positive signals and their intensities using the following formula: H-Score = ∑(pi×i), where “p_i_” represents the proportion of the positive signal pixel area/cell number, and “i” represents staining intensity. The H-Score ranges from 0 to 300, with higher values indicating a more robust overall positive intensity.

### 2.7 Protein docking with small-molecule drugs

The HERB database (http://herb.ac.cn/) was used to identify potential small-molecule drugs targeting APOD and PROC (21). The 3D protein structures of APOD and PROC were obtained from the PubChem database (https://pubchem.ncbi.nlm.nih.gov/) in SDF format and converted to PDB format using the Open Babel GUI (22). Thereafter, possible binding sites were predicted, and the software AutoDock Vina (23) and PyMOL (24) were used to identify molecules and proteins with the lowest binding free energy for further investigation.

### 2.8 Cell culture

The GC cell lines HGC-27 and SNU-216 were sourced from NHC Key Laboratory of Diagnosis and Therapy of Gastrointestinal Tumor, Gansu Provincial Hospital and maintained in a complete medium containing 10% fetal bovine serum (VivaCell, Shanghai, China), 1640 basal medium (Gibco, Shanghai, China), and 0.1% penicillin-streptomycin solution (Gibco, Shanghai, China) and cultured at 37°C with 5% CO_2_ in a constant-temperature incubator.

### 2.9 Cell Counting Kit-8 Assay

GC cells were seeded into 96-well plates and incubated overnight. Different drug concentrations were added sequentially, and the cells were incubated for an additional 48h. Next, 10 μL CCK-8 reagent (MA0218, Meilunbio, China) was added to each well, which was incubated for 2 hours. Optical density (OD) was determined at 450 nm using a microplate reader (Thermo Fisher Scientific, Massachusetts, USA). The concentration exhibiting the lowest cytotoxicity was selected for the ensuing experiments.

### 2.10 Colony formation assay

GC cells were seeded into six-well plates. The next day, the cells were incubated for approximately 10 days at 37°C in a 5% CO_2_ atmosphere after drug intervention. They were then washed with phosphate-buffered saline (PBS), fixed with 4% paraformaldehyde for 20 minutes, and stained with 1% crystal violet for 10 minutes. After rinsing with tap water, the number of cell clones was counted.

### 2.11 EdU assay

GC cells were plated in confocal petri dishes. Once the cells adhered to the dish, a complete medium containing the drug was added and incubated for 48h. Subsequently, the EdU working solution (C0075S, Beyotime, Shanghai, China) was added to the petri dishes and incubated for 2h. Afterward, the cells were fixed using 4% paraformaldehyde for 20 minutes, followed by incubation with the click reaction solution in the dark. Cells were then treated with a DAPI-containing antifade mounting medium (P0131-5ml, Beyotime, Shanghai, China), and images were captured under a fluorescence microscope.

### 2.12 Subcutaneous xenograft model

Twenty-four male nude mice (3–5 weeks old, SPF grade) were purchased from Huachuang Sino Medical Technology Co. LTD (Jiangsu, China). After a 3-day acclimatization period in an SPF-grade animal facility, a subcutaneous injection of 200 μL PBS containing 5×10^6^ HGC-27 GC cells was administered into the right axilla of each mouse using a syringe. For the Ba-treated group, intraperitoneal injections of Ba (100 mg/kg) were administered daily starting from the day of tumor cell inoculation. For the Ca-treated group, subcutaneous injections of Ca (10 mg/kg) were given every 3 days beginning on the day of tumor cell inoculation. During the experimental period, the longest and shortest diameters of the tumors were measured, and the tumor volumes were calculated using the formula: (length×width²)/2. The mice were euthanized three weeks after tumor cell inoculation, and the tumors were excised, weighed, and photographed for further analysis.

### 2.13 Wound-healing assay

The cells were seeded into six-well plates and cultured until they reached 100% confluence overnight, after which a vertical scratch was made using a pipette tip. The cells were washed three times with PBS before incubation in serum-free medium containing either the control or the drug. Cell migration was observed and photographed under an inverted microscope at 24h. The relative scratch healing rate was calculated using the following formula: (initial scratch width – scratch width at 24h)/initial scratch width.

### 2.14 Transwell assay

In the Transwell assay, 1×10^5^ GC cells were introduced into the upper chamber, which was subsequently placed in a well containing complete medium. After 24h of incubation, the chambers were removed, and the cells on the lower surface of the membrane were fixed in 4% paraformaldehyde for 20 minutes. The fixed cells were then stained with 1% crystal violet for 10 minutes. After washing with PBS, the cells on the lower surface of the membrane were photographed and counted under a microscope.

### 2.15 Statistical analysis

R (version 4.1.3) and GraphPad Prism (version 9.5) were used for statistical analysis. Student’s t-test was used for comparisons between two groups, while one-way analysis of variance (ANOVA) was used for comparisons between more than two groups. The Chi-square test was used to analyze categorical variables. Spearman’s rank correlation coefficient was used to assess correlations between variables. For survival analysis, samples were stratified into high and low expression groups based on median expression level, and statistical significance was assessed using the log-rank test. Statistical significance defined as a two-tailed p-value < 0.05. *In vitro* experiments: At least three biological and technical replicates. *In vivo* experiments: Six biological replicates and one technical replicate.

## 3 Results

### 3.1 ARID1A mutations affect TME and prognosis

**Figure 1:**
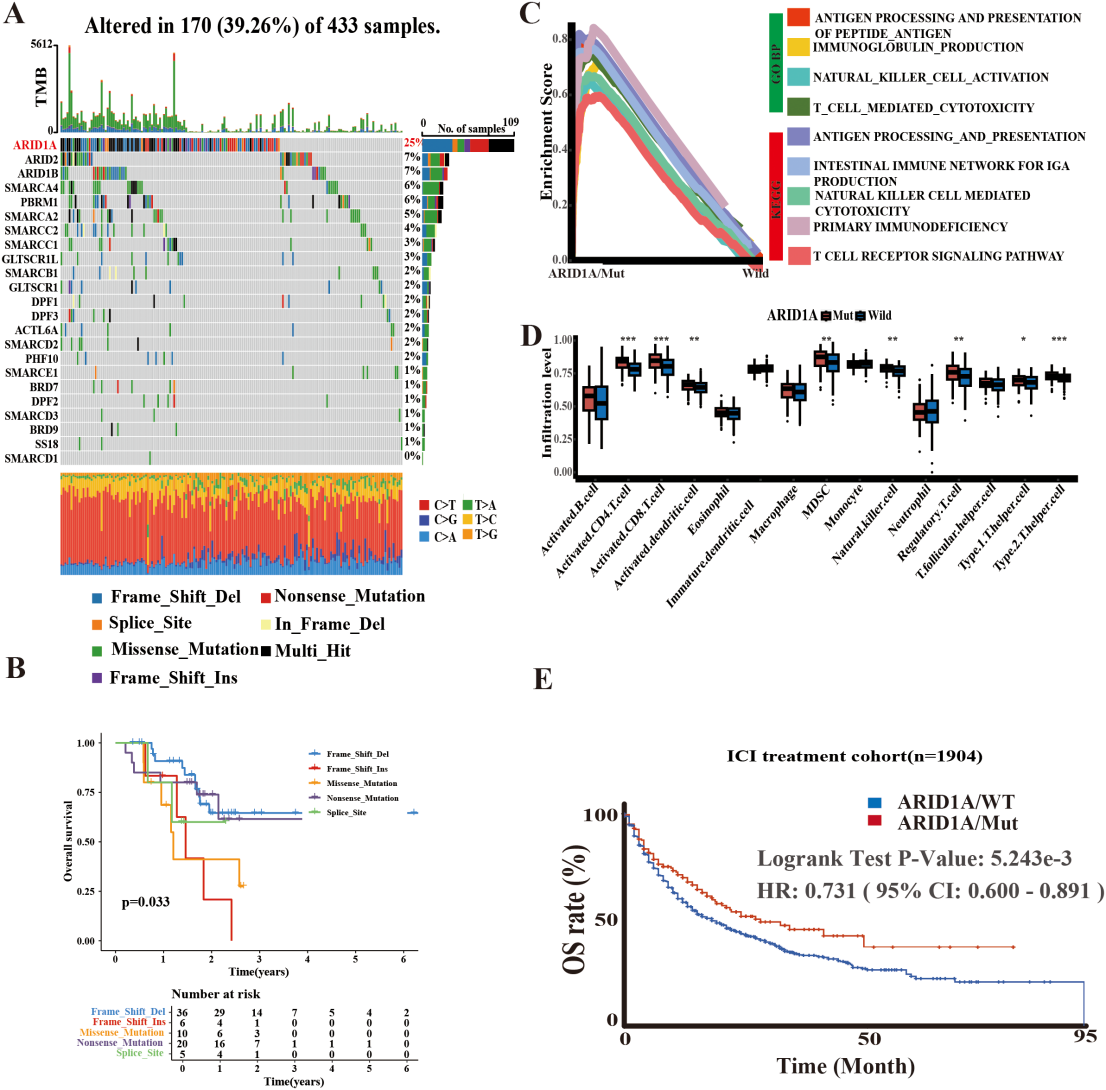
Potential significance of ARID1A mutations. **(A)** Mutation information of 23 SWI/SNF-related genes in TCGA-STAD. **(B)** Survival analysis based on ARID1A mutation types. **(C)** GSEA enrichment analysis comparing ARID1A mutant and wild-type groups. **(D)** Single-sample GSEA (ssGSEA) quantifying differences in immune cell infiltration levels between ARID1A mutant and wild-type cases. **(E)** Kaplan-Meier (KM) method illustrating the impact of ARID1A mutation status on outcomes following immune checkpoint inhibitor (ICI) therapy. *p< 0.05, **p < 0.01 and ***p< 0.001.

The SWI/SNF complex is a central component of the chromatin remodeling machinery that plays a decisive role in tumorigenesis and tumor progression. Given that mutations in 23 genes associated with the SWI/SNF complex are common genetic abnormalities in several solid tumors, including GC, we analyzed their exonic sequences in 443 cases from TCGA-STAD. Consistent with expectations, ARID1A exhibited the highest mutation frequency (25%), followed by ARID2 and ARID1B, both at 7% (**Figure 1A**). Moreover, survival analysis based on different types of ARID1A mutations revealed that patients harboring the ARID1A Frame Shift Del mutation had a better prognosis than those with Frame Shift Ins, Missense Mutation, Nonsense Mutation and Splice Site mutations (**Figure 1B**). This finding suggests the functional heterogeneity of ARID1A mutations.

To elucidate the functional implications of ARID1A mutations, we performed GSEA using gene sets from the GO and KEGG databases, stratified by ARID1A mutation status (MUT/WT). The findings indicated that the ARID1A^MUT^ group was enriched in several immune-related pathways, such as antigen processing and presentation, immunoglobulin production, and the intestinal immune network for IgA production (**Figure 1C**). Similar to GSEA result analysis, the ssGSEA algorithm revealed that ARID1A^Mut^ GC harbored a greater abundance of immune cells, including CD4^+^ and CD8^+^ T lymphocytes (**Figure 1D**).

Furthermore, survival outcomes associated with ARID1A^Mut^ were examined by reviewing immunotherapy cohorts in the cBioPortal database. The findings implied that patients harboring ARID1A^Mut^ exhibited longer survival rates following ICI therapy (**Figure 1E**), providing further evidence of the central role of ARID1A in shaping the TME and the prognosis of cancer patients.

### 3.2 Construction of a risk model based on ARID1A mutated differential prognostic immune genes

**Figure 2:**
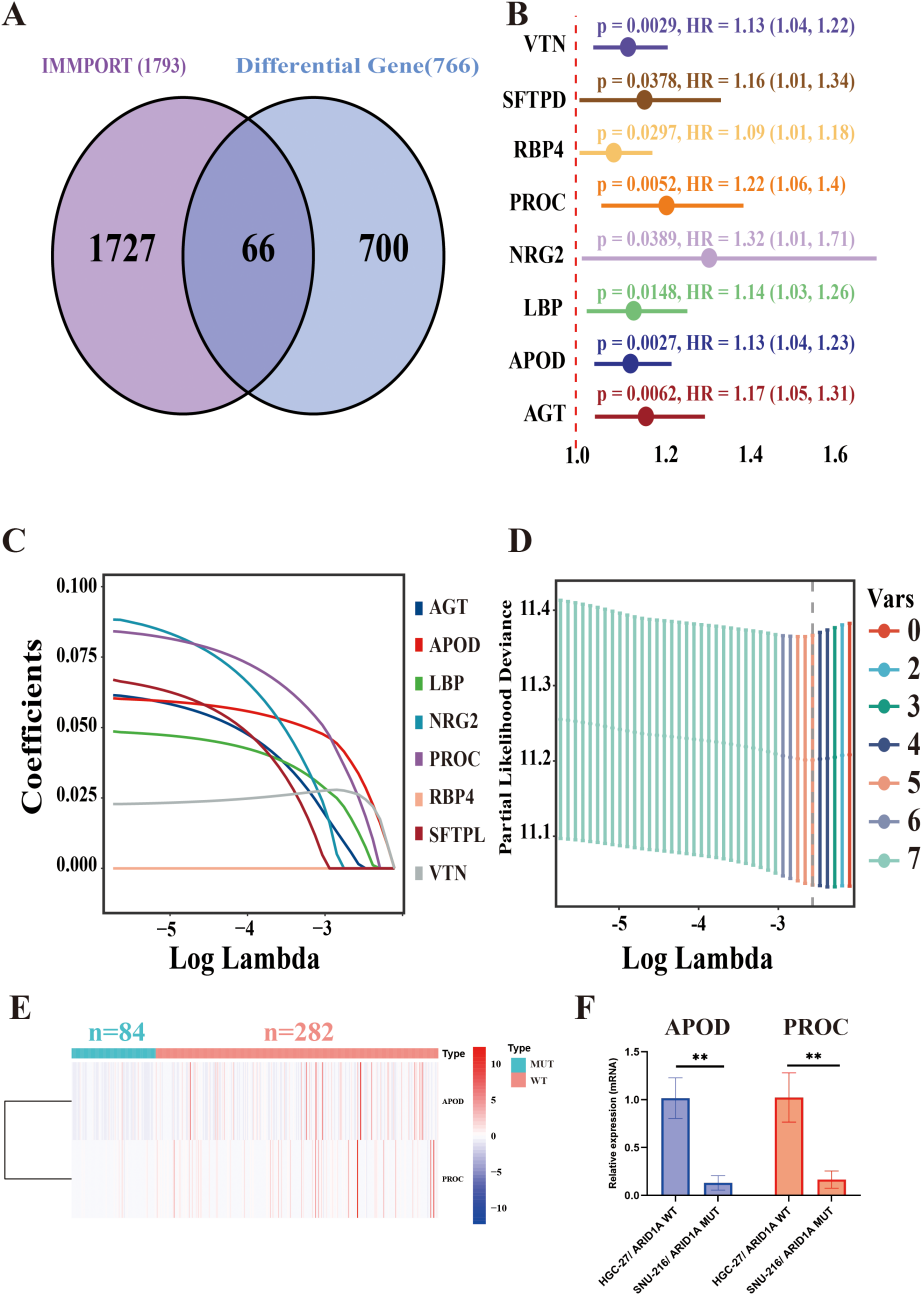
ARID1A-associated immune gene risk (ARM) model construction. **(A)** Venn diagram displaying the overlap between IMMPORT-associated immune genes and ARID1A mutation-associated differentially expressed genes. **(B)** Forest plot of eight prognostically significant immune-related genes. **(C)** LASSO coefficient profiles of the eight immune-related genes. **(D)** Selection of the optimal model parameters: The regularization path is depicted, with the dashed line indicating the minimum lambda value (lambda. min). **(E)** Heatmap of differential expression of APOD and PROC in ARID1A mutant and wild group. **(F)** APOD and PROC mRNA levels of HGC-27 and SNU-216 cell lines were analyzed via qRT-PCR. **p < 0.01.

A total of 766 DEGs were identified between the ARID1A^MUT/WT^ groups using the ‘limma’ R package. These differentially expressed genes (DEGs) were cross-referenced with the IMMPORT database, known to catalog 1,793 immune-related genes. This approach yielded 66 immune-related genes associated with ARID1A mutations (**Figure 2A**).

To construct a model for assessing prognosis, the TCGA-STAD cohort was initially partitioned into the train- (n = 253) and test groups (n = 84) at a 3:1 allocation ratio. Clinical characteristics were comparable between the two groups (**Supplementary Table 2**). In the training group, univariate Cox regression analysis identified eight genes, namely VTN, SFTPD, RBP4, PROC, NRG2, LBP, APOD, and AGT, that were significantly associated with a poor prognosis (*p <* 0.05) (**Figure 2B**). Following this, the number of genes was reduced using the Lasso method. Initially, the regression coefficient trends for eight independent variables were analyzed as log lambda (log λ) values increased (**Figure 2C**). Afterward, the five genes with the minimum partial likelihood of deviance were selected for subsequent risk modeling (**Figure 2D**). Finally, a prognostic model was developed using APOD and PROC, two of the five immune-related genes, to construct the ARID1A mutation-associated risk model (ARM). The ARM risk score was calculated as follows: ARM risk score = (0.104558626103791 × APOD) + (0.150191346083483 × PROC). **Figure 2E** showed that both APOD and PROC were expressed at lower levels in GC patients harboring ARID1A^Mut^ (n = 84) compared to those with wild-type ARID1A (n = 282). The qRT-PCR data further confirmed that the expression levels of APOD and PROC were significantly lower in the ARID1A^Mut^ GC cell line SNU-216 compared to the ARID1A^WT^ GC cell line HGC-27 (*p* < 0.01) (**Figure 2F**).

### 3.3 ARM effectively predicts the prognosis of GC patients

**Figure 3:**
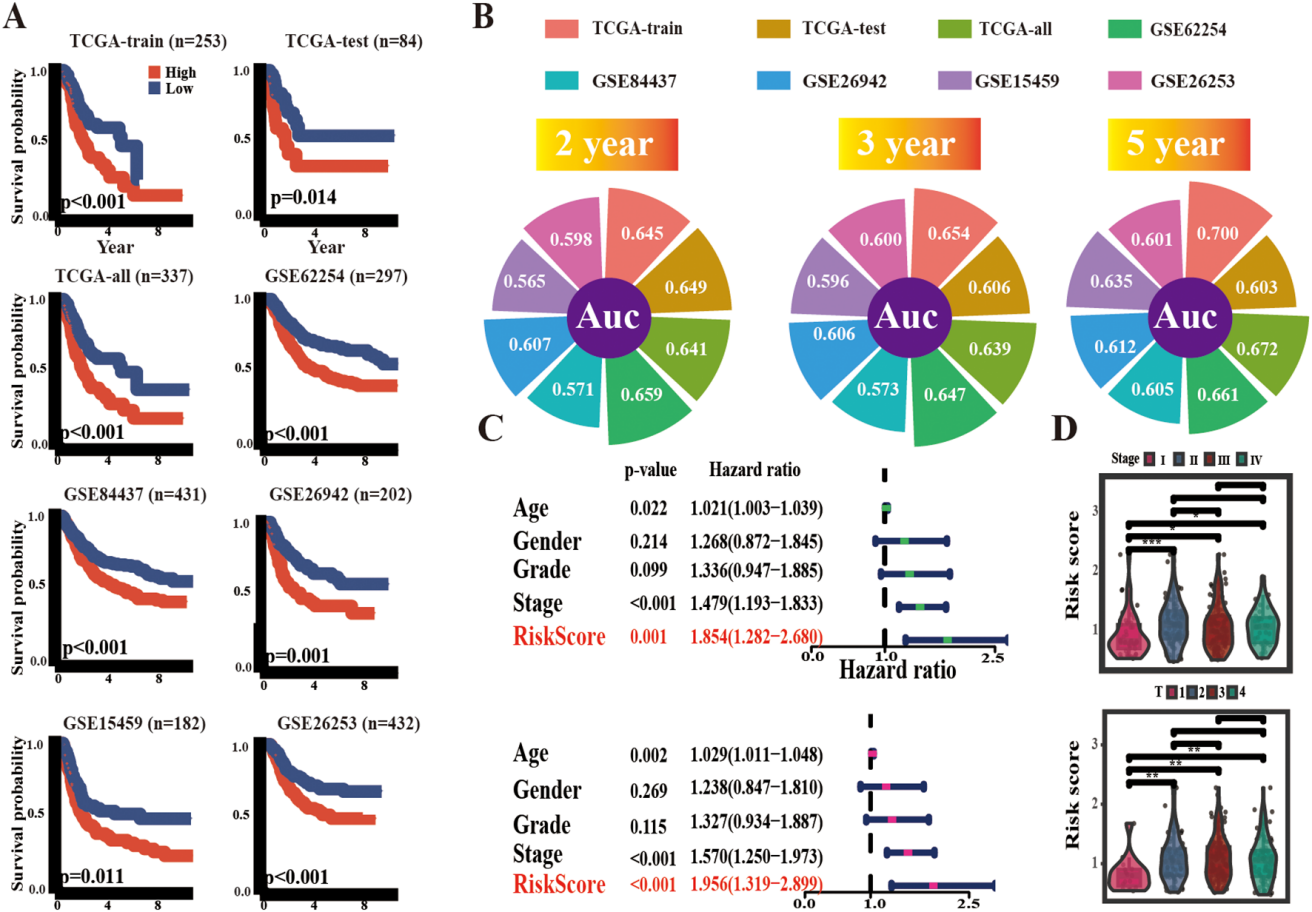
Prognostic potential of ARM. **(A)** KM survival curves delineating overall survival (OS) across multiple cohorts, including TCGA-train, TCGA-test, TCGA-all, GSE62254, GSE84437, GSE26942, and GSE15459, as well as recurrence-free survival (RFS) in GSE26253. **(B)** 2,3,5 years AUC values of ARM in different cohorts. **(C)** Univariate and multivariate regression analyses of ARM risk scores. **(D)** Distribution of ARM risk scores across TNM stages (top panel) and T stages (bottom panel). * for *p* < 0.05, ** for *p* < 0.01, and *** for *p* < 0.001.

To determine the predictive value of ARM, a median ARM risk score was calculated to stratify the cohorts into high-risk and low-risk groups. In contrast to low-risk patients, high-risk patients had poorer overall survival (OS) across the TCGA-train, TCGA-test, and all groups, as determined by KM analysis (TCGA-train: *p* < 0.001; TCGA-test: *p* = 0.014; TCGA-all: *p* < 0.001). Then, the ARM risk score formula was employed to categorize five independent GEO GC cohorts (GSE62254, GSE84437, GSE26942, GSE15459, and GSE26253) into high- and low-risk categories. This consistently revealed that the high-risk group exhibited an unfavorable prognosis (**Figure 3A**). Further evaluation of ARM reliability was conducted through time-dependent receiver operating characteristic (ROC) curve analysis, with area under the curve (AUC) values computed at 2/3/5-year intervals for both the TCGA-STAD and GEO cohorts. In the TCGA-train group, the AUC values were 0.645, 0.654, and 0.700, respectively. In contrast, the AUC values were 0.649, 0.606, and 0.603 in the TCGA-test group and 0.641, 0.639, and 0.672 in the TCGA-all group, respectively. AUC values for the GEO datasets were as follows: GSE62254: 0.659, 0.647 and 0.661; GSE84437: 0.571, 0.573 and 0.605; GSE26942: 0.607, 0.606 and 0.612; GSE15459: 0.565, 0.596 and 0.635; GSE26253: 0.598, 0.600 and 0.601 (**Figure 3B**). Additionally, using t-distributed stochastic neighbor embedding (t-SNE) and principal component analysis (PCA), distinct clustering and differentiation between the low/high-risk populations were observed (**Supplementary Figure 1**).

Ultimately, conventional clinical characteristics, such as gender, age, tumor grading, and staging, were incorporated for univariate and multivariate Cox regression analyses, and the ARM risk score exhibited independent prognostic characteristics in TCGA-STAD (**Figure 3C**). Furthermore, the risk scores for stages II-IV were higher than those in stage I. Likewise, risk scores for stages T2–4 were higher than those in stage T1 (**Figure 3D**).

### 3.4 ARM alters the TME in GC

**Figure 4:**
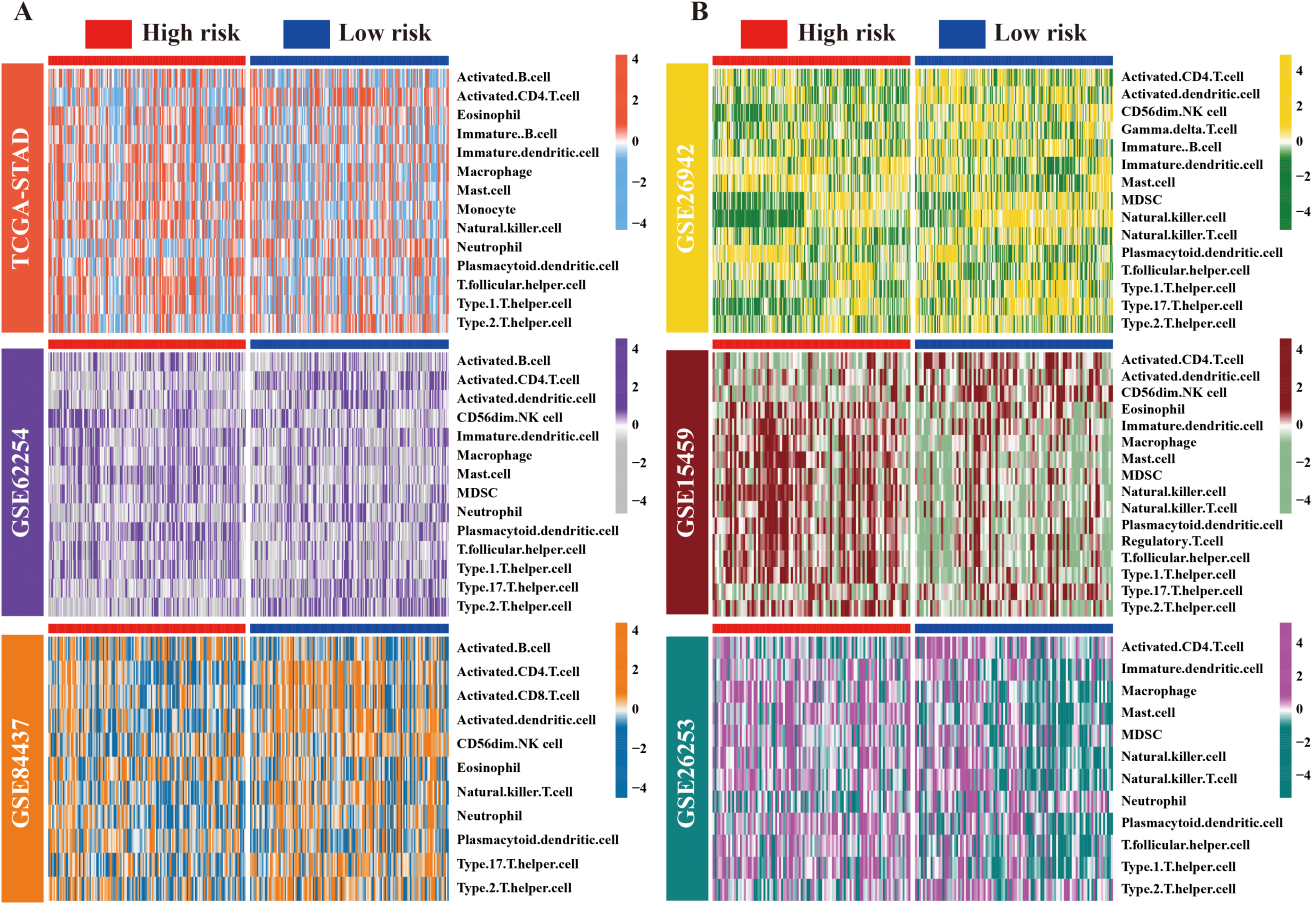
Landscape of immune cell distribution in high/low-risk ARM populations. **(A)** Heat maps depicting variations in immune cell infiltration levels across the high- and low-risk groups within the TCGA, GSE62254, and GSE84437 cohorts, presented in a top-to-bottom orientation. **(B)** Variations in immune cell infiltration levels in the GSE26942, GSE15459, and GSE26253 cohorts (from top to bottom).

To examine the possible causes of the prognostic differences between the high- and low-risk groups, the ssGSEA algorithm was applied to analyze TME characteristics in TCGA-STAD and five GEO cohorts. Importantly, significant differences were observed in TME components among groups with varying risk levels. Specifically, across all cohorts, the low-risk group was associated with a consistent increase in the abundance of activated CD4^+^ T cells. In contrast, the abundance of plasma dendritic cells was lower. Moreover, a marked increase was noted in the abundance of macrophages and mast cells in the high-risk group, except in the GSE62254 cohort. Furthermore, variations in the infiltration levels of other types of immune cells, such as T helper type 1 and 2 (Th1/2), activated CD8^+^ T cells, natural killer T cells (NKT), and neutrophils exhibited abnormal patterns in at least one of the GC cohorts (**Figures 4A, B**). We posit that alterations in the TME may have contributed to the prognostic changes.

### 3.5 ARM can serve as a reference tool for assessing ICI treatment effectiveness

**Figure 5:**
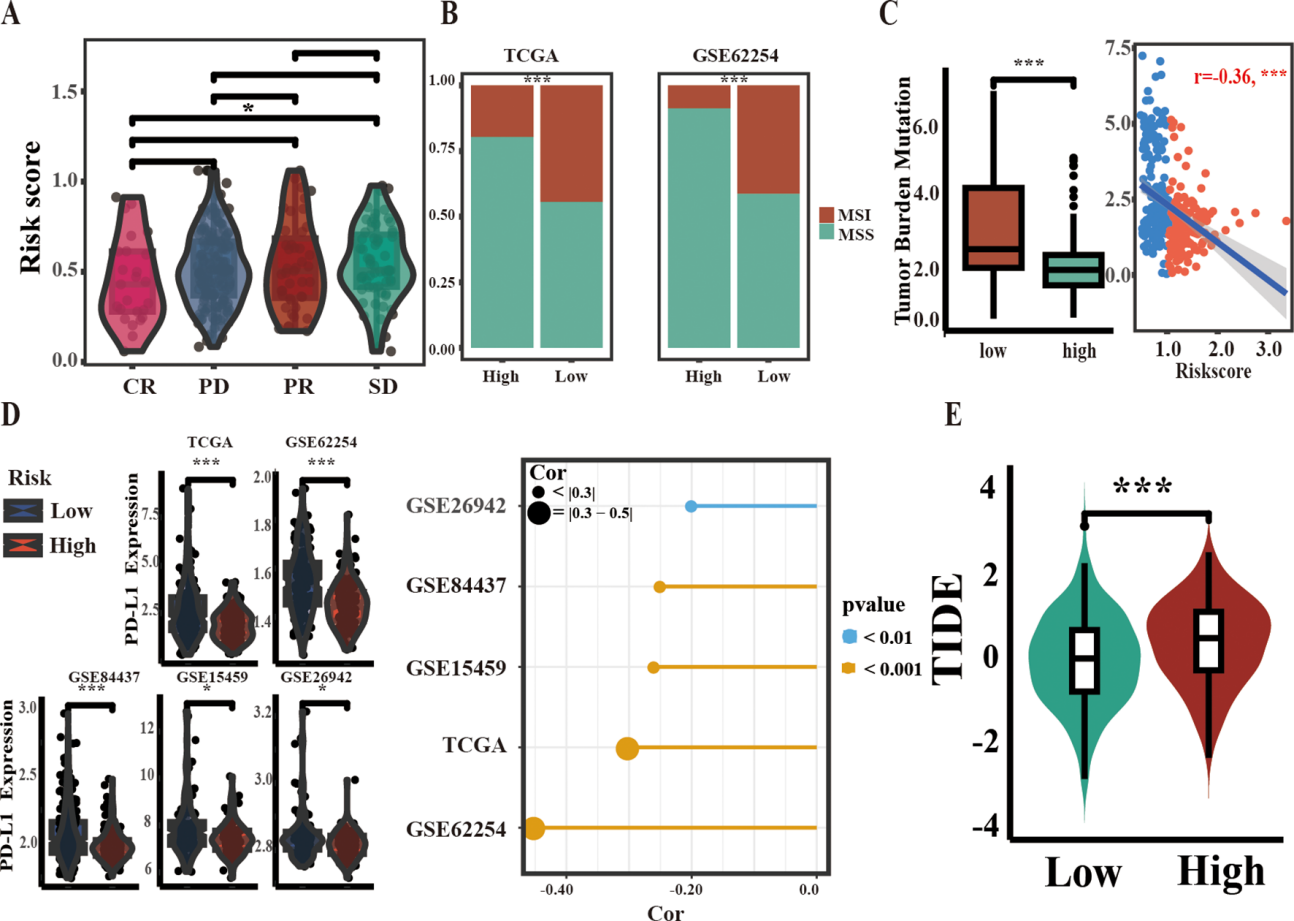
Correlation between ARM and biomarkers of ICI therapy efficacy. **(A)** Stratification of ARM risk scores within four PD-L1 response groups in the IMvigor210 cohort. **(B)** Comparison of MSI percentages across different groups within two independent cohorts using the chi-square test. **(C)** Comparison of TMB values between the two groups (left) and Spearman correlation analysis of ARM risk scores and TMB values in TCGA-STAD (right). **(D)** Comparison of PD-L1 mRNA levels across groups (left), with analysis of their correlation with risk scores using Spearman’s method (right). **(E)** Variations in TIDE scores between the low-risk and high-risk populations of ARM. Complete response [CR], partial response [PR], stable disease [SD], progressive disease [PD]; * for *p* < 0.05; *** for *p* < 0.001.

Microsatellite instability (MSI), tumor mutational burden (TMB), and CD274 (PD-L1) expression are recognized as emerging markers for evaluating the efficacy of ICI therapies (25, 26). In the context of the IMvigor210 PD-L1 treatment cohort, an analysis of risk score disparities among patients stratified into the complete response (CR), partial response (PR), progressive disease (PD), and stable disease (SD) groups demonstrated that the CR group had lower risk scores than the SD group. However, comparisons among other groups did not reveal statistically significant differences (**Figure 5A**). In both the TCGA and GSE62254 cohorts, chi-square tests indicated a greater prevalence of MSI GC in the low-risk group compared to the high-risk group (**Figure 5B**). Similarly, TMB values and PD-L1 expression were higher in low-risk individuals, and risk scores were negatively correlated with both parameters (**Figures 5C, D**). TIDE score, an indicator of immune response, suggested that lower scores were correlated with favorable outcomes in tumor immunotherapy. In this study, a lower TIDE score was observed in the low-risk TCGA group. (**Figure 5E**). Taken together, these results suggest that the low-risk group is more likely to respond favorably to ICI therapy.

### 3.6 ARM accurately predicts the prognosis of GC patients from Hainan General Hospital

**Figure 6:**
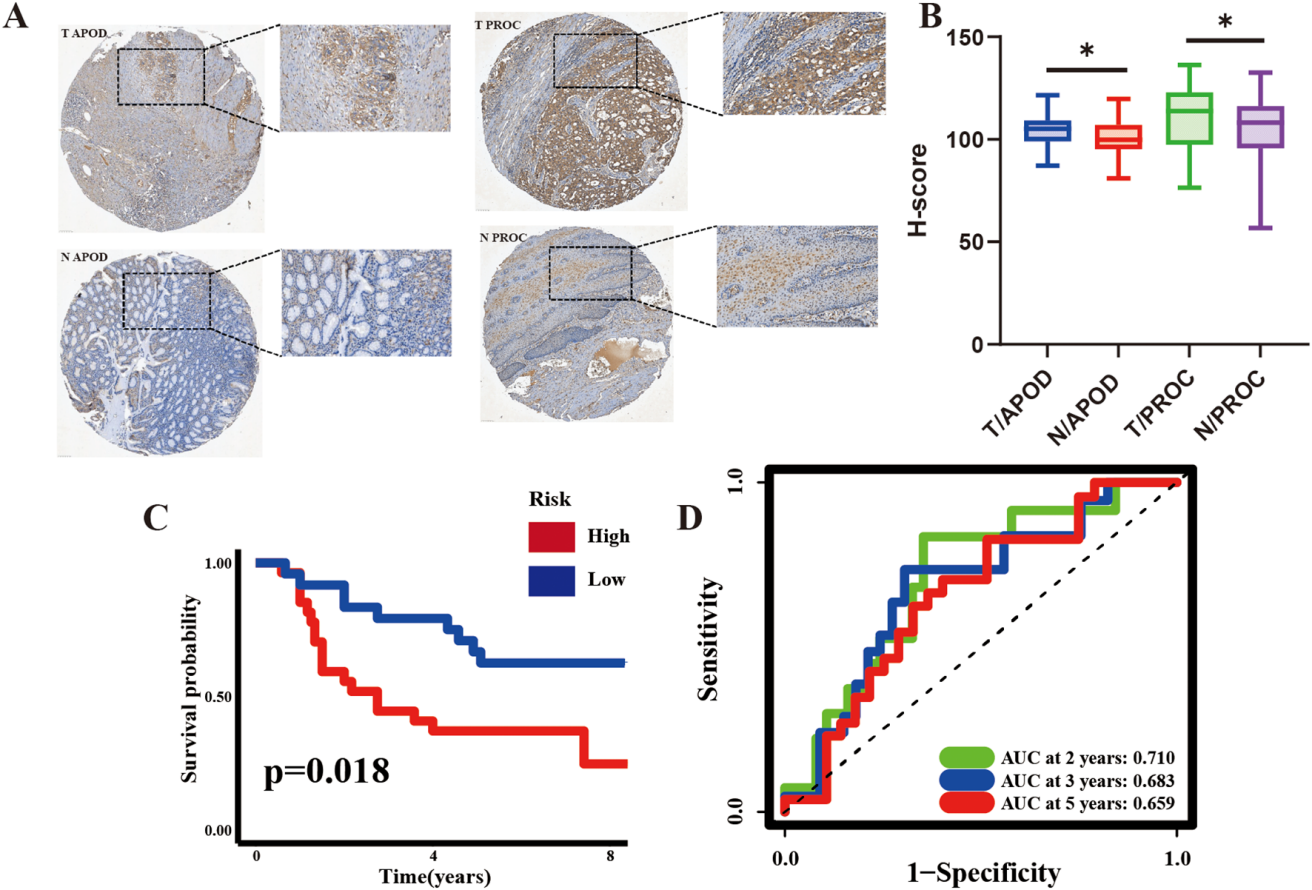
Prognostic Significance of ARM in 55 GC patients Evaluated by Immunohistochemistry (IHC). **(A)** Representative IHC images of APOD (left) and PROC (right) in GC tissues and corresponding adjacent non-cancerous tissues. **(B)** H-score for APOD and PROC expression in GC and adjacent non-cancerous tissues. **(C, D)** KM curves and time-dependent ROC curves for the Hainan GC cohort. H-scores, indicative of the protein expression intensity, were computed using Visiopharm software; *p <0.05. T, tumor; N, adjacent non-cancerous tissues.

To investigate the potential of ARM in predicting GC prognosis, GC and matched paracarcinoma tissues from 55 patients (clinical details are listed in **Supplementary Table 3**) from Hainan General Hospital were subjected to immunohistochemical analysis. The immunohistochemical images of APOD and PROC expression patterns in tissues showed a predominant cytoplasmic localization for both APOD and PROC proteins (**Figure 6A**).

The H-score, a semi-quantitative metric derived from the intensity of IHC antibody staining as an indirect measure of protein expression levels, was applied. The results demonstrated that APOD and PROC expression was significantly higher in GC tissues compared to the adjacent non-malignant tissues (**Figure 6B**). KM analysis further demonstrated that the high-risk group had a significantly poorer prognosis than the low-risk group. Additionally, AUC values (2-year: 0.697, 3-year: 0.673, 5-year: 0.648) highlighted the strong prognostic ability of ARM (**Figures 6C, D**), implying that it could effectively predict GC prognosis at both the mRNA and protein levels.

### 3.7 Molecular docking of small molecules targeting ARM-related immune genes inhibits GC cell proliferation, migration, and invasion

**Figure 7:**
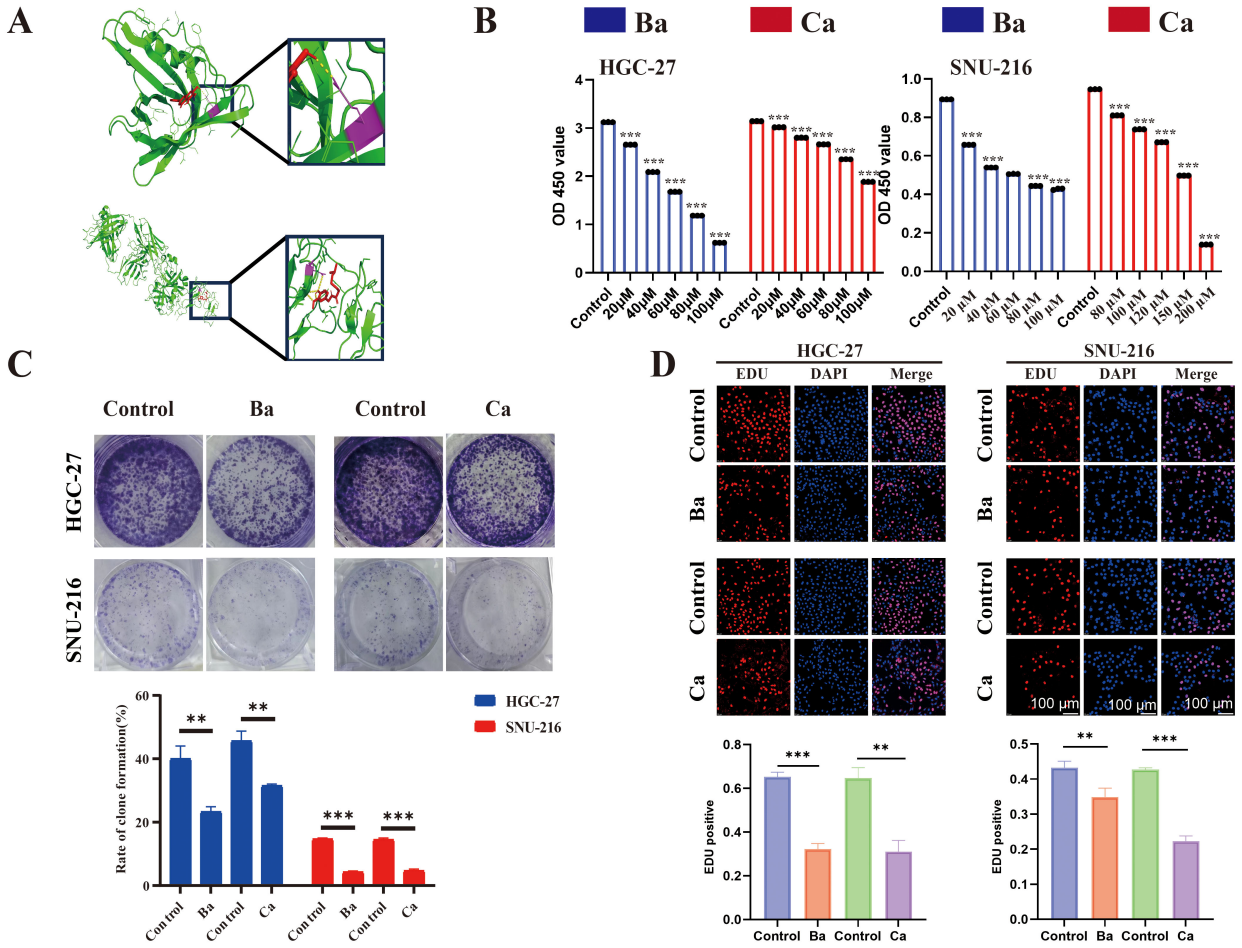
Molecular docking and drugs on GC cell proliferation. **(A)** Three-dimensional (3D) view of the optimal conformations for APOD and baicalein (up), PROC, and capsaicin (down). Green areas indicate target proteins, whereas red areas represent small molecules, yellow areas denote hydrogen bonds between the protein and the small molecule, and purple areas indicate binding sites. **(B)** CCK8 assay was performed to assess the proliferative ability of GC cells (HGC-27 and SNU-216) after drug treatment. **(C)** A colony formation assay was performed to assess the colony-forming ability of GC cell lines after drug treatment. **(D)** EdU assay for GC cells after drug intervention and matched quantitative analysis results. Ba, baicalein; Ca, capsaicin. ** for *p* < 0.01, and *** for *p* < 0.001. Error bars indicate SD; The error bars are short due to the low variance.

To identify therapeutic drugs targeting APOD and PROC, the HERB database was utilized. Ba and Ca emerged as promising small-molecule candidates that selectively target APOD and PROC. Subsequent molecular docking experiments, conducted using AutoDock Vina software, yielded compelling results: a binding energy of -7.9 kcal/mol was observed for the APOD-Ba interaction, while the PROC-Ca interaction exhibited a binding energy of -6.8 kcal/mol, indicating a substantial potential for binding affinity between these protein-ligand pairs. To further elucidate the nature of these interactions, PYMOL software was employed to construct three-dimensional visualizations of the protein-ligand complexes. Moreover, the binding sites for APOD-Ba and PROC-Ca, were delineated (**Figure 7A**). The results collectively suggested that both APOD-Ba and PROC-Ca interactions exhibit high binding specificity and strength.

**Figure 8:**
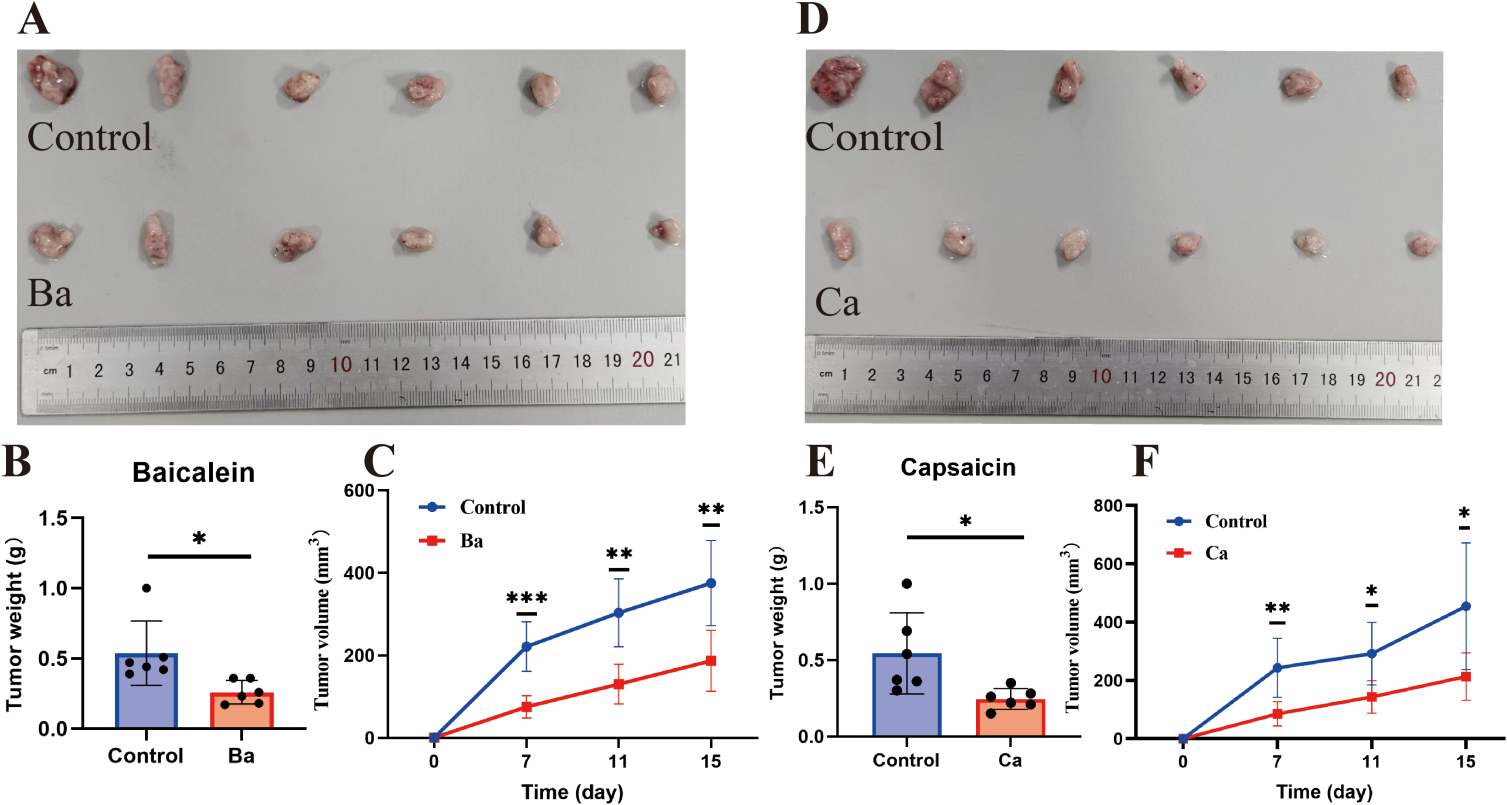
Ba and Ca inhibit subcutaneous tumorigenicity in nude mice. **(A-C)** From left to right, real images of tumors in the Control and Ba groups, histograms of tumor weight differences between the two groups, and volume curves at different time points (days 7, 11, and 15). **(D–F)** Control and Ca groups. Note: *p < 0.05. **p < 0.01; ***p < 0.001. Ba, baicalein; Ca, capsaicin.

The CCK8 assay showed that Ba and Ca exerted concentration-dependent inhibition of the proliferative ability of GC cells after 48h of treatment (**Figure 7B**). Then, the drug concentrations (Ba, 40μM; Ca, 80μM) with minimal effects on cell viability were chosen for the subsequent experiments. The results of the clone formation assay showed that the clonogenic ability of cells in the drug-treated groups was significantly reduced (**Figure 7C**). The EdU assay also revealed a lower EdU-positive rate in the Ba and Ca groups compared with that in the matched control group (**Figure 7D**). The above results conjointly demonstrated that Ba and Ca can inhibit the proliferation of GC cells *in vitro*. Further, *in vivo* experiments demonstrated that the Ba-treated group exhibited a significantly reduced mean tumor weight and a markedly smaller mean tumor volume at multiple time points (days 7, 11, and 15) compared to the control group (**Figures 8A-C**). Likewise, the Ca-treated group also displayed robust antitumor effects, with its mean tumor weight and mean tumor volume being significantly lower than those of the control group at all measured time points (**Figures 8D-F**). These findings further substantiate the potential of Ba and Ca in suppressing the growth of GC cells *in vivo*, thereby providing promising insights for the development of novel therapeutic strategies for GC treatment or the retardation of disease progression.

**Figure 9:**
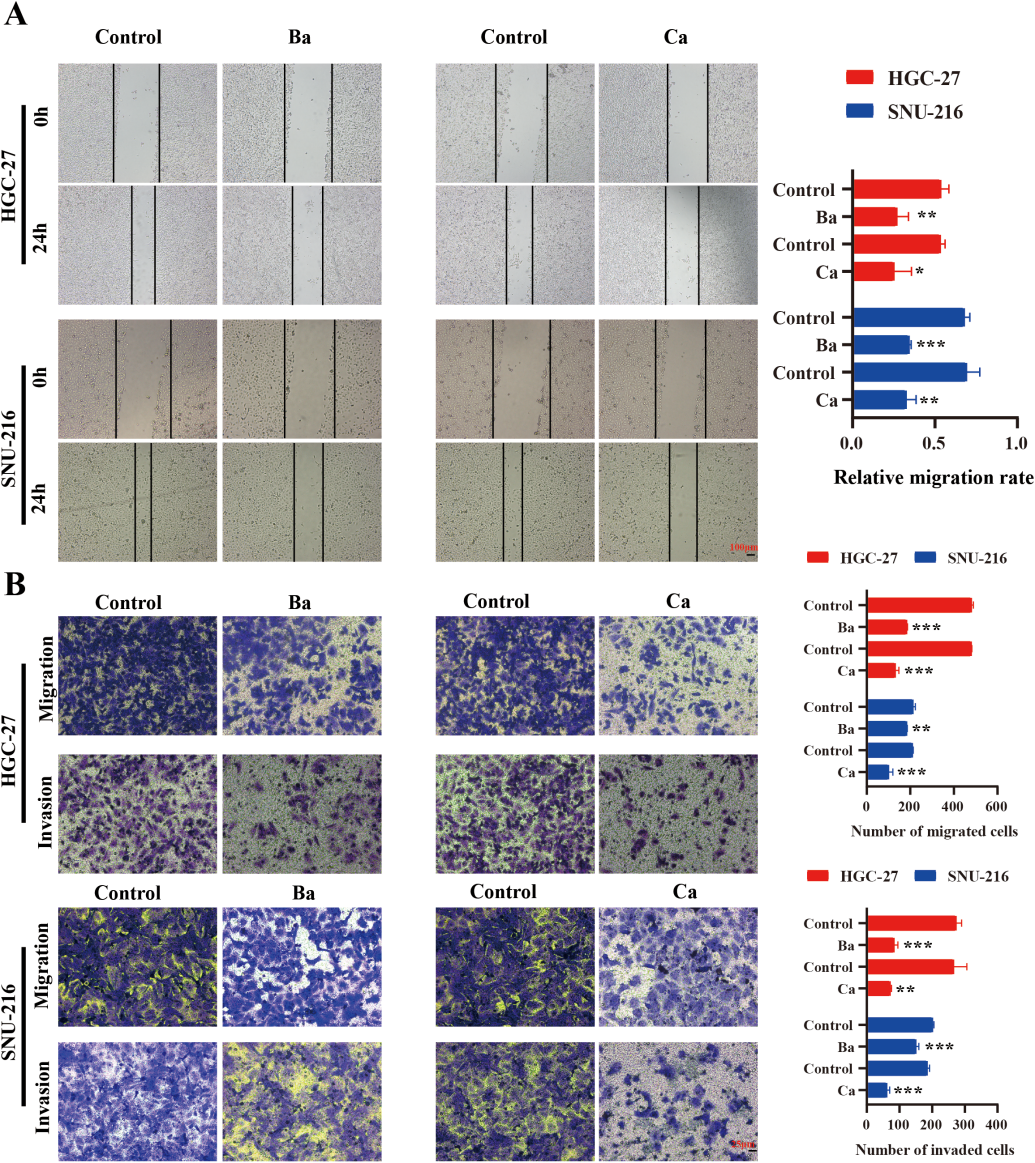
Correlation between drugs and the migratory and invasive abilities of GC cells. **(A)** Analysis of alterations in the migratory capability of GC cells 24h post-drug intervention utilizing a wound healing assay. **(B)** Differences in the migratory and invasive abilities of GC cells between drug and non-drug groups were assessed after 24h via the Transwell assay. Ba, baicalein; Ca, capsaicin. * for *p* < 0.05, ** for *p* < 0.01, and *** for *p* < 0.001.

Metastasis is a key feature of tumor cells (27). To explore the effect of Ba and Ca on the migratory and invasive abilities of GC cells *in vitro*, a wound healing assay was performed. The results revealed that the relative migratory ability in the Ba and Ca groups was lower compared to the control group (**Figure 9A**). Similarly, after 24h, the results of the Transwell assay validated that the number of migrating and invading cells was lower in the Ba and Ca groups compared to the control group (**Figure 9B**). In conclusion, these results provide preliminary evidence supporting the antitumorigenic effects of Ba and Ca.

## 4 Discussion

ARID1A is frequently mutated across various types of cancers, with loss-of-function mutations being the most prevalent (28). Dysfunction of ARID1A can lead to immune escape of tumor cells by upregulating PD-L1. On the other hand, immune cell infiltration abundance is higher in cancer tissues by regulating the expression of immune genes (25, 29, 30). This phenomenon confers a molecular signature associated with enhanced immunotherapeutic efficacy, which may explain the improved prognosis observed in ARID1A^Mut^ cancer populations undergoing ICI therapy.

Herein, the high mutation frequency of ARID1A was associated with a stronger immune-activating phenotype and a higher abundance of CD8^+^ and CD4^+^ T cells in GC tissues. In addition, ARID1A^Mut^ cancer patients had a better prognosis following ICI treatment. ARID1A^Mut^-related differentially expressed immune genes might have significant clinical value in predicting prognosis and response to immunotherapy.

To develop a prognostic model for more precise prognosis assessment, APOD and PROC, two ARID1A mutation-associated prognostic immune genes were selected to construct the prognostic model (ARM), which demonstrated satisfactory prognostic predictive ability in TCGA datasets and five independent GC patient cohorts from the GEO database. More importantly, it successfully predicted the prognosis of GC patients from Hainan General Hospital. Overall, these findings underscore the utility and reliability of ARM as a prognostic assessment tool for GC.

APOD overexpression was correlated with poor prognosis in GC and breast cancer (31, 32). Notably, a model incorporating APOD and nine other genes demonstrated considerable prognostic value for GC (33). APOD belongs to the broader family of apolipoproteins and participates in lipid metabolism. It can transform normal stromal cells into COL11A1^+^ tumor-associated fibroblasts, which is associated with tumor progression and prognosis (34). In addition, the PROC gene encodes protein C, a component of the protein C anticoagulant system that plays an essential role in maintaining normal anticoagulant function. Studies demonstrated that aberrant expression of PROC can impair coagulation processes and consequently facilitate tumor cell migration and invasion (35). Bioinformatics analysis identified PROC as a gene significantly associated with CD4^+^ T cells, with immunohistochemical evaluations of 139 GC cases identifying it as a high-risk factor (36). Herein, APOD and PROC were highly expressed in GC tissues compared to non-cancerous tissues. Moreover, Ba and Ca were identified as potential small-molecule compounds of ARM-related genes that exert antitumorigenic effects. Ba and Ca have been widely reported to inhibit cancer cell proliferation and metastasis and promote cell death (37–42). However, relevant studies in GC are limited. In the present study, these two drugs influenced the proliferative, migratory, and invasive abilities of GC cells, highlighting their utility in the treatment of GC patients or for delaying disease progression.

Alterations in TME have been reported to be strongly associated with cancer progression and prognosis (43). Therefore, variations in immune characteristics between the low- and high-risk groups were examined. The results revealed a higher infiltration level of tumor-suppressive immune cells, such as activated CD4^+^ T cells whereas tumor-promoting immune cells, such as mast cells and macrophages, exhibited lower infiltration levels. These variations in TME components between the high- and low-risk groups might explain the disparities in prognosis between these groups.

ARM also demonstrated potential utility in evaluating immunotherapy responses, as confirmed in an ICI treatment cohort comprising more than 300 cases. This enhanced predictive capability may be attributed to the low-risk group exhibiting higher TMB, a greater proportion of MSI patients, and increased PD-L1 expression levels. Notably, the ARM-risk score is negatively correlated with TMB values and PD-L1 expression levels; the latter was confirmed in multiple GC cohorts. These findings highlights the potential of our model as a robust tool for identifying GC patients who are more likely to respond to immunotherapy.

The other six prognostic genes identified in this research have also been recognized for their potential as cancer prognostic markers and their involvement in the regulation of cancer cell proliferation, invasion, migration, and chemoresistance. For example, VTN knockdown in GC cell lines reduced proliferative and invasive capacities, with VTN identified as a poor prognostic factor in 156 GC patients (44). Meanwhile, SFTPD has been identified as a potential prognostic marker for lung and colorectal cancer (45). The RBP4/RhoA/Rock1 axis has been found to regulate the proliferation, migration, and invasion of ovarian cancer cells (46), while NRG2 was identified as a prognostic marker for GC and breast cancer (47, 48) and LBP as a prognostic marker for GC (49). Several studies identified AGT as a prognostic marker for GC (50–52), with one study demonstrating that AGT was implicated in the regulation of chemosensitivity and proliferation and metastatic abilities of GC cells (53). These studies emphasized the critical role of these genes in tumorigenesis.

Nevertheless, several limitations of this study merit acknowledgment. ARID1A plays a critical role in chromatin remodeling and gene expression. However, this study did not establish a direct causal relationship between ARID1A mutations and the altered expression of the two immune genes included in the ARM. The mechanism by which ARID1A mutations influence interactions between the immune system and GC remains to be elucidated. Secondly, additional research is necessary to validate the robustness and clinical applicability of the ARM. Finally, further investigation is required to elucidate the biological functions and roles of these two immune genes in tumorigenesis.

## 5 Conclusion

In summary, a prognostic risk model (ARM) was developed herein based on two immune genes associated with ARID1A mutations. ARM effectively predicted the prognosis of GC patients using data from the TCGA and GEO datasets, as well as our hospital cohort. Furthermore, low-risk individuals exhibited a more favorable response to ICI therapy. These findings collectively provide a novel approach for assessing clinical prognosis in GC. Moreover, Ba and Ca were identified as potential small-molecule compounds for GC treatment.

## Data Availability

The original contributions presented in the study are included in the article/**Supplementary Material**. Further inquiries can be directed to the corresponding author/s.
