# Correction: Development and validation of an ARID1A-related immune genes risk model in evaluating prognosis and immune therapeutic efficacy for gastric cancer patients: a translational study

**DOI:** 10.3389/fimmu.2025.1637370

**Published:** 2025-06-13

**Authors:** Jiangtao Zhang, Jingting Li, Shangfeng Yang, Xiaoyan Tang, Chunze Wang, Jiaxing Lin, Qiancheng Chen, Hui Xu, Yuanyuan Ma, Xiaoling Gao

**Affiliations:** ^1^ The Clinical Laboratory Center, Hainan General Hospital, Hainan Affiliated Hospital of Hainan Medical University, Haikou, Hainan, China; ^2^ Hainan Medical University, Haikou, China; ^3^ Second Department of Critical Care Medicine, Xi’an Daxing Hospital, Shanxi, China

**Keywords:** ARID1A, gastric cancer, molecular docking, immune gene risk model, prognosis

In the published article, there was an error in affiliation(s) 1. Instead of “The Clinical Laboratory Center, Hainan General Hospital, Hainan Affiliated Hospital of Hainan, Medical University, Haikou, Hainan, China.”, it should be “The Clinical Laboratory Center, Hainan General Hospital, Hainan Affiliated Hospital of Hainan Medical University, Haikou, Hainan, China.”.

In the published article, there was an error in the legend for **Figure 7** as published. The Y-axis label in the statistical graph of **Figure 7D** should have the percentage symbol removed, and **Figure 7B** has been updated accordingly. The corrected legend appears below.

**Figure 7:**
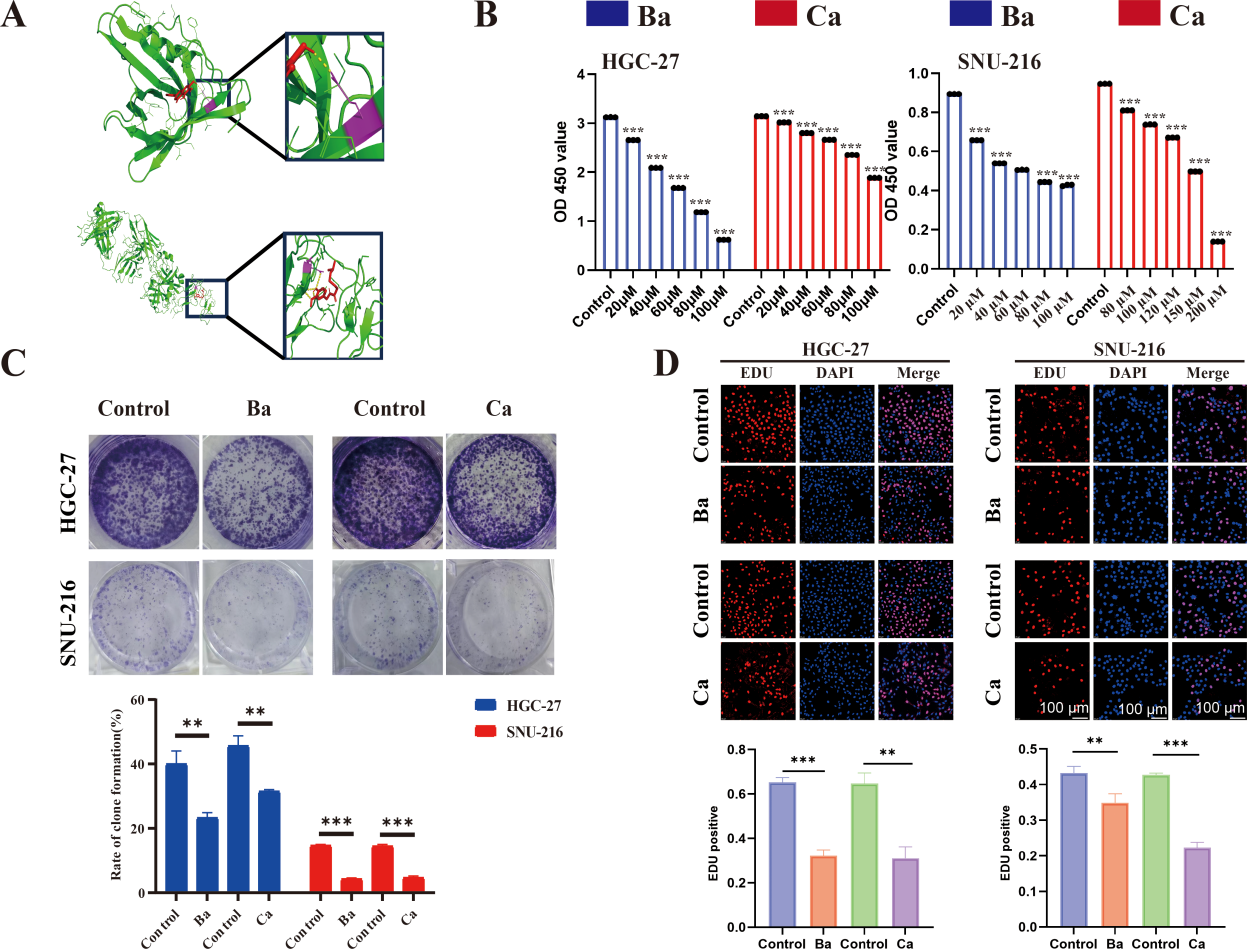
Molecular docking and drugs on GC cell proliferation. **(A)** Three-dimensional (3D) view of the optimal conformations for APOD and baicalein (up), PROC, and capsaicin (down). Green areas indicate target proteins, whereas red areas represent small molecules, yellow areas denote hydrogen bonds between the protein and the small molecule, and purple areas indicate binding sites. **(B)** CCK8 assay was performed to assess the proliferative ability of GC cells (HGC-27 and SNU-216) after drug treatment. **(C)** A colony formation assay was performed to assess the colony-forming ability of GC cell lines after drug treatment. **(D)** EdU assay for GC cells after drug intervention and matched quantitative analysis results. Ba, baicalein; Ca, capsaicin. ** for p < 0.01, and *** for p < 0.001. Error bars indicate SD; The error bars are short due to the low variance.

The authors apologize for this error and state that this does not change the scientific conclusions of the article in any way. The original article has been updated.

