# Supplementary material for: Development and validation of an ARID1A-related immune genes risk model in evaluating prognosis and immune therapeutic efficacy for gastric cancer patients: a translational study
Source: Front Immunol. 2025 Apr 28;16:1541491. doi: 10.3389/fimmu.2025.1541491 (PMC12096169; doi:10.3389/fimmu.2025.1541491)
Supplement: Supplementary file 1 [file DataSheet1.docx]

**Development and validation of an ARID1A-related immune genes risk model in evaluating prognosis and immune therapeutic efficacy for gastric cancer patients: a translational study**

**Jiangtao Zhang^1, 2†^, Jingting Li^1, 2†^, Shangfeng Yang^2†^, Xiaoyan Tang^1, 2^, Chunze Wang^1, 2^, Jiaxing Lin^1, 2^, Qiancheng Chen^2^, Hui Xu^1^, Yuanyuan Ma^3^, Xiaoling Gao^1, 2, *^**

^1^ The Clinical Laboratory Center, Hainan General Hospital (Hainan Affiliated Hospital of Hainan Medical University), Haikou, Hainan, China.

^2^Hainan Medical University, Haikou, Hainan, China.

^3^ Second Department of Critical Care Medicine, Xi'an Daxing Hospital, Shanxi, China

***Corresponding author:** Dr. Xiaoling Gao, e-mail:[gaoxl008@hotmail.com](mailto:gaoxl008@hotmail.com). The Medical Laboratory Center, Hainan Affiliated Hospital of Hainan Medical University, 19 Xiuhua Road, Xiuying District, Haikou, Hainan Province 570311, China.

**^†^**These authors have contributed equally to this work

Supplementary Fig 1 and Table 1-3


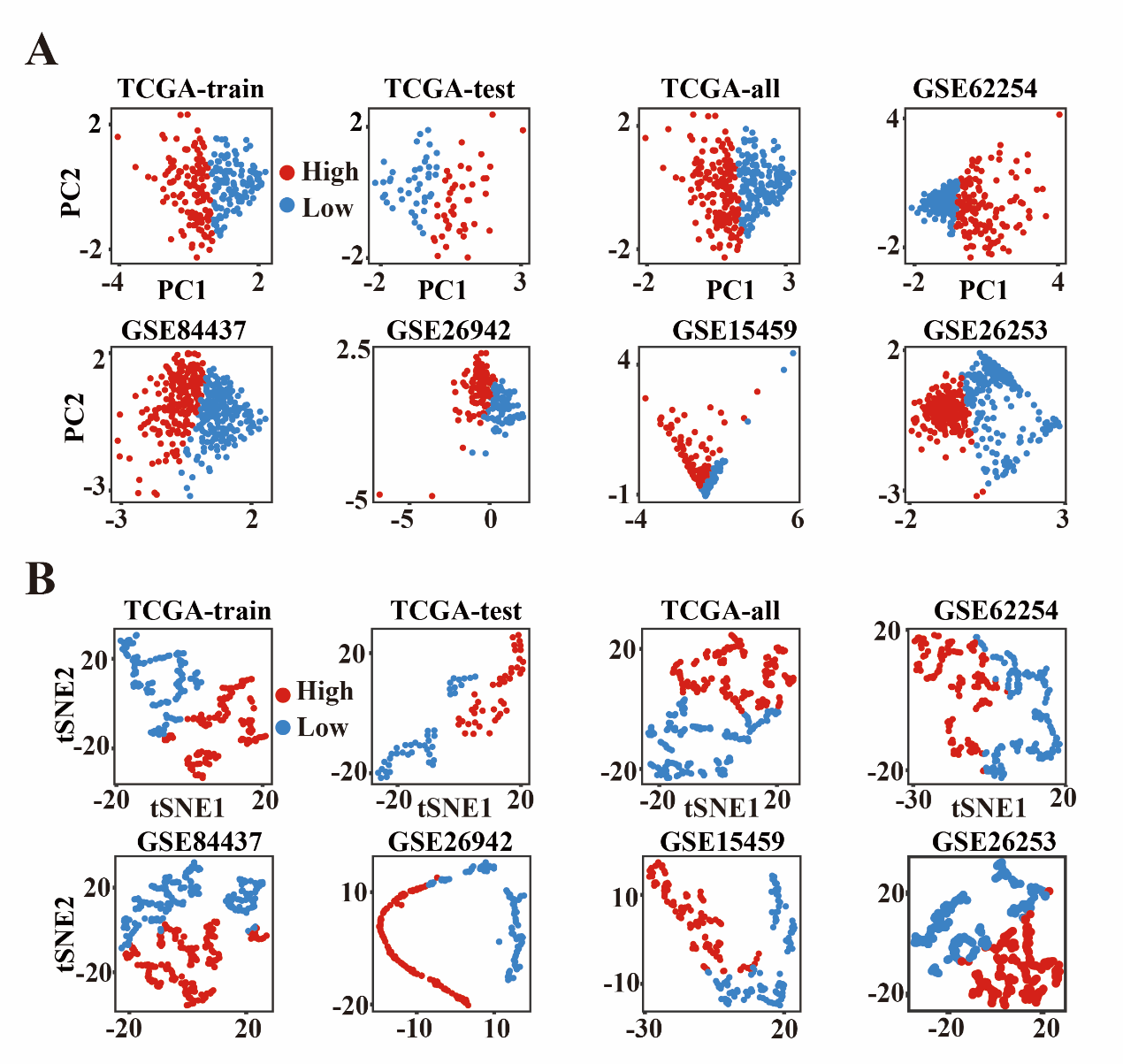


**Supplementary Fig 1. Analyzing risk group distribution information in different cohorts using dimensionality reduction techniques**. (A) Principal component analysis (PCA) and (B) t-distributed stochastic neighbor embedding (t-SNE) plot of distribution pattern high- and low-risk groups.

**Supplementary Table 1. primer sequences**

| **Gene name** | **sequences** |
| --- | --- |
| GAPDH-F | GCACCGTCAAGGCTGAGAAC |
| GAPDH-R | TGGTGAAGACGCCAGTGGA |
| APOD-F | AACCAGGAGTTGAGAGCTGATG |
| APOD-R | GTACGGTGCCGATGGCATA |
| PROC-F | GCACACCAGCTCCTCTTGAC |
| PROC-R | CCAGAAGGCCAGTGTGTCAT |

**Supplementary Table 2. Clinical characteristics of TCGA-train and TCGA-test groups**

| Variables | Type | Total | Test | Train | P value |
| --- | --- | --- | --- | --- | --- |
| Age | <=65 | 153(45.4%) | 36(42.86%) | 117(46.25%) | 0.6164 |
|  | >65 | 181(53.71%) | 48(57.14%) | 133(52.57%) |  |
|  | unknow | 3(0.89%) | 0(0%) | 3(1.19%) |  |
| Gender | FEMALE | 119(35.31%) | 30(35.71%) | 89(35.18%) | 1 |
|  | MALE | 218(64.69%) | 54(64.29%) | 164(64.82%) |  |
| Grade | G1 | 9(2.67%) | 4(4.76%) | 5(1.98%) | 0.3646 |
|  | G2 | 120(35.61%) | 30(35.71%) | 90(35.57%) |  |
|  | G3 | 199(59.05%) | 47(55.95%) | 152(60.08%) |  |
|  | unknow | 9(2.67%) | 3(3.57%) | 6(2.37%) |  |
| Stage | Stage I | 45(13.35%) | 12(14.29%) | 33(13.04%) | 0.9629 |
|  | Stage II | 107(31.75%) | 26(30.95%) | 81(32.02%) |  |
|  | Stage III | 137(40.65%) | 32(38.1%) | 105(41.5%) |  |
|  | Stage IV | 34(10.09%) | 9(10.71%) | 25(9.88%) |  |
|  | unknow | 14(4.15%) | 5(5.95%) | 9(3.56%) |  |
| T | T1 | 15(4.45%) | 6(7.14%) | 9(3.56%) | 0.2149 |
|  | T2 | 74(21.96%) | 19(22.62%) | 55(21.74%) |  |
|  | T3 | 156(46.29%) | 32(38.1%) | 124(49.01%) |  |
|  | T4 | 88(26.11%) | 26(30.95%) | 62(24.51%) |  |
|  | unknow | 4(1.19%) | 1(1.19%) | 3(1.19%) |  |
| M | M0 | 303(89.91%) | 73(86.9%) | 230(90.91%) | 0.3032 |
|  | M1 | 22(6.53%) | 8(9.52%) | 14(5.53%) |  |
|  | unknow | 12(3.56%) | 3(3.57%) | 9(3.56%) |  |
| N | N0 | 99(29.38%) | 25(29.76%) | 74(29.25%) | 0.3749 |
|  | N1 | 91(27%) | 19(22.62%) | 72(28.46%) |  |
|  | N2 | 68(20.18%) | 22(26.19%) | 46(18.18%) |  |
|  | N3 | 68(20.18%) | 15(17.86%) | 53(20.95%) |  |
|  | unknow | 11(3.26%) | 3(3.57%) | 8(3.16%) |  |

**Supplementary Table 3. Clinicopathological information of 55 Hainan GC patients**

| **Variable** | **Hainan GC cohort (n = 55)** | |
| --- | --- | --- |
| **Age** | n | % |
| >65 | 37 | 67.27 |
| ≤65 | 18 | 32.73 |
|  |  |  |
| **Gender** |  |  |
| Male | 37 | 67.27 |
| Female | 18 | 32.73 |
|  |  |  |
| **Grade** |  |  |
| Ⅰ-Ⅱ | 3 | 5.45 |
| Ⅱ | 11 | 20.00 |
| Ⅱ-Ⅲ | 11 | 20.00 |
| Ⅲ | 18 | 32.73 |
| unknow | 12 | 21.82 |
|  |  |  |
| **Stage** |  |  |
| I | 15 | 27.27 |
| II | 13 | 23.64 |
| III | 24 | 43.64 |
| IV | 3 | 5.45 |
|  |  |  |
| **T** |  |  |
| 1 | 8 | 14.54 |
| 2 | 13 | 23.64 |
| 3 | 12 | 21.82 |
| 4 | 22 | 40.00 |
|  |  |  |
| **N** |  |  |
| 0 | 23 | 41.82 |
| 1 | 10 | 18.18 |
| 2 | 14 | 25.45 |
| 3 | 8 | 14.55 |
|  |  |  |
| **M** |  |  |
| 0 | 52 | 94.55 |
| 1 | 3 | 5.45 |
